# Analysis of Sequence Conservation at Nucleotide Resolution

**DOI:** 10.1371/journal.pcbi.0030254

**Published:** 2007-12-28

**Authors:** Saurabh Asthana, Mikhail Roytberg, John Stamatoyannopoulos, Shamil Sunyaev

**Affiliations:** 1 Division of Genetics, Brigham and Women's Hospital, Harvard Medical School, Boston, Massachusetts, United States of America; 2 Computational Biology Group, Institute of Mathematical Problems in Biology, Russian Academy of Sciences, Pushchino, Russia; 3 Department of Genome Sciences, University of Washington, Seattle, Washington, United States of America; University of Toronto, Canada

## Abstract

One of the major goals of comparative genomics is to understand the evolutionary history of each nucleotide in the human genome sequence, and the degree to which it is under selective pressure. Ascertainment of selective constraint at nucleotide resolution is particularly important for predicting the functional significance of human genetic variation and for analyzing the sequence substructure of *cis*-regulatory sequences and other functional elements. Current methods for analysis of sequence conservation are focused on delineation of conserved regions comprising tens or even hundreds of consecutive nucleotides. We therefore developed a novel computational approach designed specifically for scoring evolutionary conservation at individual base-pair resolution. Our approach estimates the rate at which each nucleotide position is evolving, computes the probability of neutrality given this rate estimate, and summarizes the result in a Sequence CONservation Evaluation (SCONE) score. We computed SCONE scores in a continuous fashion across 1% of the human genome for which high-quality sequence information from up to 23 genomes are available. We show that SCONE scores are clearly correlated with the allele frequency of human polymorphisms in both coding and noncoding regions. We find that the majority of noncoding conserved nucleotides lie outside of longer conserved elements predicted by other conservation analyses, and are experiencing ongoing selection in modern humans as evident from the allele frequency spectrum of human polymorphism. We also applied SCONE to analyze the distribution of conserved nucleotides within functional regions. These regions are markedly enriched in individually conserved positions and short (<15 bp) conserved “chunks.” Our results collectively suggest that the majority of functionally important noncoding conserved positions are highly fragmented and reside outside of canonically defined long conserved noncoding sequences. A small subset of these fragmented positions may be identified with high confidence.

## Author Summary

The structure of the human genome remains largely unknown, including which parts of the genome are functionally relevant and which parts are “junk.” The availability of genomic sequence from a large number of mammals allows a more detailed exploration of this structure, using comparison of related sequences from different species to identify portions of the genome that have remained unchanged, conserved by the action of natural selection, and thus likely to be functionally significant. To date, most efforts focused on localizing the functional fraction of the human genome have been based on identifying contiguous stretches of positions conserved in multiple species. Here, we present an analysis that is based instead on a single-position measure of conservation called SCONE. Our analysis suggests that the majority of conserved and putatively functional positions are highly fragmented and lie outside contiguous regions of conserved sequence. A subset of these fragmented positions may be identified based on local clustering.

## Introduction

Comparative sequence analysis has had a major impact on molecular biology and genetics. Comparison of the sequences of protein-coding genes between multiple species has enabled prediction of gene function [1], identification of protein domains [2], prediction of functional amino acid residues [3,4], and detection of signals of natural selection at the level of whole genes [5] and individual codons [6,7]. Inferring non-neutral sequence elements in the human genome is of considerable interest even without a specific a priori hypothesis concerning their possible functional role(s). On a general level, for example, sequence conservation may considerably inform human genetic studies seeking to identify allelic variants associated with disease phenotypes, particularly in noncoding regions [8]. The effect of human SNPs at the level of molecular function and phenotype depends on the importance of the individual nucleotide position, whereas the information of the sequence region as a whole is not necessarily relevant. For example, about half of human SNPs within protein coding genes are represented by synonymous variants, which are likely to be of limited importance, even though they are embedded within highly conserved exonic sequences. In addition, a subset of individual nucleotides conserved in four mammalian genomes were shown to be under selective pressure [9]. A position-specific measure of selective constraint is therefore highly suitable for analysis of positions that are polymorphic within the human population.

Several algorithms have been developed for detection and scoring of sequence conservation in the context of a multispecies sequence alignment. However, to date these approaches have been applied almost exclusively to detect discrete regions with elevated average sequence conservation that typically extend for up to hundreds of contiguous bases [10–14]. Such regions encompass canonical coding exons, as well as so-called “conserved noncoding sequences” that presumably result from purifying selection, and are thereby indicative of functional importance [15,16].

Recently, comparative genomic sequence of unprecedented depth has been generated by sequencing of multiple mammalian and other vertebrate genomes orthologous to 1% of the human genome defined by the ENCODE regions [17,18]. Several alignment techniques have been applied to construct multiple sequence alignments within ENCODE regions [18]. These alignments have in turn been subjected to analysis with existing sequence conservation detection algorithms, including phastCons[10], GERP [11], and BinCons [13]. The conserved regions identified by these analyses show statistically significant overlap with experimentally identified coding and noncoding functional elements. However, the majority of experimentally characterized noncoding functional elements fall outside of currently delineated conserved regions, and, conversely, most conserved regions were located outside of experimentally detected elements [18]. The fact that many functional elements reside in noncoding regions that do not exhibit uniformly high conservation is perhaps not surprising given that binding sites for transcriptional factors that mediate many biological processes are quite plastic evolutionarily [19]. Conversely, many individual nucleotides located outside of well-defined conserved regions exhibit sequence conservation across multiple species. Such conservation may be due to mere chance or, for a certain fraction of these nucleotides, may reflect their importance for fitness and hence function. The aforementioned observations emphasize the need for higher resolution methods for analysis of evolutionary conservation within functional elements and generally across the genome.

Here we develop an approach for analyzing sequence conservation at the individual base-pair level, with an aim toward correlating conservation with human genetic variation and with functional genomic annotations. We present a new probabilistic conservation score, SCONE (Sequence Conservation Evaluation). SCONE provides conservation scores for individual nucleotide positions, and can be applied to predict continuous sequence regions with an elevated level of conservation.

We apply SCONE to the study of annotated functional elements and human sequence polymorphism. We focus on the statistical distribution of position-specific conservation scores rather than on the bulk overlap between conserved regions and functional features. It is clear from the outset that the power to detect conservation at the single base-pair resolution is limited, even when comparing multiple species [20]. We surmount this obstacle by deriving considerable statistical power from combined analysis of numerous individual nucleotide positions from many genomic regions. While this analysis does not allow us to detect individual functional positions accurately, we can show that, collectively, a subset of noncontiguous individual positions are important. A key advantage of the analysis of the distribution of position-specific scores is that it is unbiased with respect to the pattern of conservation along a given sequence region. SCONE thus has the potential to analyze putative functional elements in which the conservation signal is not homogeneous or manifested by exon-like contiguous conserved stretches.

We report herein on the relationship between sequence conservation, functional sequence elements, and human allelic variation, as revealed by single-nucleotide conservation analysis.

## Results

**Figure 1:**
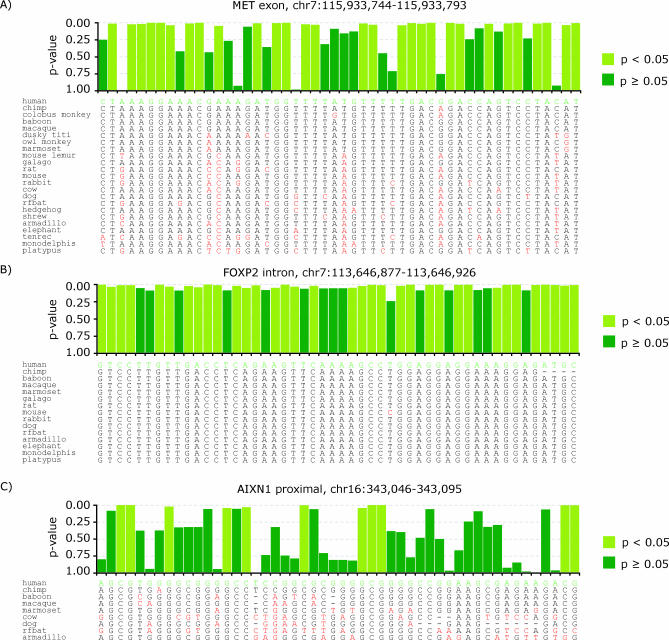
Examples of SCONE *p*-Value Scores for Coding (A), Highly Conserved Noncoding (B), and Nonconserved Regions Positions likely to be conserved (*p* < 0.05) are in light green; other positions are dark. Below each plot is the portion of the multiple sequence used to generate scores for each sequence region. Deviations from human sequence (green) are indicated in red. (A) A portion of an exon from the MET gene (chr7:115,933,744–115,933,793). The pattern of conserved positions is indicative of the triplet structure of the genetic code. (B) A highly conserved intronic sequence in the FOXP2 gene (chr7:113,646,877–113,646,926). (C) An intergenic region near the AXIN1 gene (chr16:343,046–343,095) showing little overall conservation, but containing a significant number of individually conserved positions.

SCONE provides an estimate of the rate at which a given position (column) in a multiple sequence alignment is evolving and a probability (*p*-value) of neutrality for that position, based on a model of neutral evolution. We used SCONE to score conservation in all alignable human bases using the phylogenetic tree and multiple sequence alignments (generated by the TBA alignment program [21]) made available by the ENCODE Multiple Sequence Alignment group [18]. Figure 1 shows an example of SCONE scores. Though positions were human-referenced, we excluded human sequence from conservation analysis to avoid ascertainment biases with regard to the study of human SNPs (see Methods). Positions containing fewer than two aligned sequences were also excluded from scoring. Despite these limitations, SCONE scores are available for 27.6 out of 30 Mbases of ENCODE sequences (92%). We examined the distribution of *p*-values for SCONE scores in putative neutral sites (see Methods). As *p*-values for SCONE scores correspond to the hypothesis of neutrality, their distribution in neutral positions should be uniform. On average, the distribution strongly resembles a uniform distribution (Figure S1A), showing that the model of evolution employed by SCONE is in general agreement with the observed pattern of evolution.

SCONE presents both an estimate of evolutionary rate (a measure of the intensity of constraint) and a *p*-value for a null hypothesis of neutrality based on the rate. Though ascertaining rate (and thereby constraint) is in many instances the more appropriate measure to use in the pursuit of functional positions, reliability of conservation detection using the rate estimate varies with respect to sequence coverage (Figure S1B). *p*-Values, on the other hand, control fraction of neutrally evolving positions scored as conserved independently of sequence coverage.

SCONE is available to download as a stand-alone program for UNIX operating systems at http://ika.bwh.harvard.edu/scone/. All SCONE scores used in this analysis may be obtained via the UCSC Genome Browser, at http://genome.ucsc.edu/ENCODE/.

### Conservation in Mammals and Human Polymorphism

Analysis of population sequence polymorphism is an effective and widely used tool for detecting the influence of ongoing or recent selection. Sites experiencing purifying selection will tend to show a decrease in the density of polymorphism and average heterozygosity, as well as a shift in allele frequencies toward more rare derived (nonancestral) alleles. We hypothesized that sites adjudged to be constrained by SCONE would evince ongoing purifying selection, which should in turn affect the distribution of population polymorphism. Differences in allele frequency distributions between positions under strong constraint and unconstrained positions, both inside and outside of contiguous conserved sequence regions, would thus indicate the functional significance of those positions.

We employed the most comprehensive SNP dataset available for these regions, produced by the International Haplotype Map project [22]. The HapMap project resequenced ten 500 kb ENCODE regions (total 5 Mb) in 48 unrelated individuals from four separate population sets (Yoruba, Han Chinese, Japanese, and CEPH). Subsequent genotyping was performed in each population on the basis of this SNP discovery and SNPs in the dbSNP database; this is likely to introduce biases toward frequent SNPs (i.e., those most likely to be shared between populations) and artificially reduce the apparent fraction of rare SNPs. We chose to rely on the parental subset of the Yoruba population from Ibadan, Nigeria (YRI), for our SNP analysis, which, after filtering out SNPs in CpG positions, included a total of 13,490 SNPs in ten ENCODE regions. In all of our analysis, we ignored SNPs within coding regions, since selective effects on coding SNPs are comparatively well-studied.

**Figure 2:**
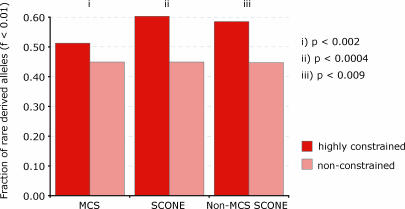
Rare Derived Allele Frequency in Conserved versus Nonconserved Sites Positions are partitioned according to (i) ENCODE MCS elements for all ENCODE positions, (ii) SCONE conservation score for all ENCODE positions, and (iii) SCONE conservation score for all ENCODE positions outside of MCS elements. *p*-Values are calculated using Fisher's exact test.

We detect a significant difference (*p* < 0.0004, Fisher exact test) in the fraction of rare derived alleles (Figure 2) between conserved (SCONE *p*-value < 0.005, Fisher exact test) and nonconserved noncoding positions. The higher fraction of rare derived alleles in conserved (slowly evolving) positions indicates that these positions are experiencing purifying selection. Because allele frequency distributions are unaffected by mutation rate heterogeneity, our results suggest that this effect is due to sites that are evolving slowly due to selection rather than merely due to chance. For comparison, we examined the allele frequency distribution in noncoding conserved sequence regions, using the ENCODE multispecies conserved sequence (MCS) element set to define contiguous conserved elements. These were defined on the basis of agreement between at least two out of three regional conservation scores (phastCons, BinCons, and GERP) that identify regions of sequence with elevated average conservation. The shift in allele frequency distributions is stronger for SCONE-conserved positions than it is for MCS elements (*p* < 0.05, Fisher exact test), suggesting that these positions are either enriched for functional positions compared to MCS elements, or are on average under stronger selection.

We employed a simple model of evolution that assumes constant population size and no demographic events to estimate the average heterozygous selection coefficient (*s*) for functional noncoding positions that best explains the observed shift in allele frequencies between SCONE conserved and nonconserved positions. We assumed, based on the false discovery rate in these positions (see Methods), that 61.6% of SCONE conserved positions are functional. We estimate *s* in the range of 10^−4^–10^−3^.

An excess of low-frequency alleles in conserved regions was reported in several earlier studies [23–25]. The main question pertinent to the analysis of position-specific conservation is whether the majority of deleterious alleles within a population reside in conserved regions, or whether individually conserved positions not incorporated into longer conserved elements are also under purifying selection. To address this question, we examined the distribution of allele frequencies in positions outside of MCS elements. After partitioning these positions according to their SCONE rate estimates (as above), we were able to detect a significant difference (*p* < 0.009) in rare derived allele frequency between high- and low-scoring positions. This strong shift may be an indication that a significant subset of functional positions lie outside of MCS elements [9], and that a greater portion of functional positions may be identifiable via the contribution of position-specific analysis than can be found through the identification of conserved elements alone. This suggests that a search for phenotypically important human genetic variation should not be limited to conserved regions, and information on the conservation level of individual base pairs is of importance for prioritizing SNPs in studies of genetics of specific human phenotypes.

### Conservation in Functional Features

Population genetic analysis indicates that a significant fraction of functional positions lies outside MCS elements. It is natural to seek confirmation of this fact by inquiring whether these positions coincide with identifiable regulatory and other functional elements, and whether we may observe a similar distribution of conserved positions and MCS elements with regard to annotated functional regions.

In addition to a highly accurate annotation of protein coding genes, the ENCODE project has produced large-scale identification of transcribed regions, a composite of putative sequence-specific binding sites, and regions with significantly increased histone modification (EIGRs) likely to be involved in transcription regulation, and DNase I Hypersensitive sites (DHSs), which are heavily validated markers of human *cis*-regulatory sequences [18].

A subset of transcribed regions identified by hybridization of RNA to tiling genomic DNA microarrays consist of nonprotein coding transcribed regions of unclear functional significance. Analysis of conservation may therefore potentially support functionality of such regions, or may enlighten the current debate concerning background transcription in the human and other large genomes [26]. Promoters represent a specific type of regulatory element, whereas EIGR and DHS tracks are more generic in nature and may therefore encompass a spectrum of functional elements with differing regulatory roles.

**Figure 3:**
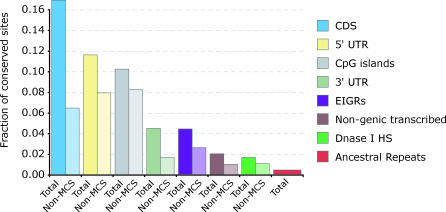
Conservation in Various Functional Classes For each functional class, the fraction of positions with SCONE *p*-value ≤ 0.005 is shown, both including (dark) and excluding (light) positions falling within MCS elements. Ancestral repeats are included as a control.

We therefore analyzed the distribution of SCONE *p*-values in all of the above types of functional elements, as well as CpG islands, a well-known sequence-based marker of functional elements. We found all categories of functional elements to exhibit statistically significant deviation from the uniform neutral distribution. The deviation of all classes of functional regions from the neutral distribution was explained almost entirely by a subset of positions with low SCONE *p*-values. The excess of conserved individual positions in functional regions compared to putatively neutral regions (ancestral repeats) is shown in Figure 3.

In many classes of functional elements, much of the signal of conservation falls outside of MCS elements. With the exception of coding sequences and 3′ UTRs, a majority of individually conserved positions do not fall within MCSs. The pattern of conservation differs markedly across different functional features; even a high overall level of conservation (as in CpG islands) does not imply that these putatively functional positions can be easily grouped into functional elements. The true proportion of functional positions may thus be underrepresented by requiring them to be grouped into windows.

Transcribed regions and EIGRs show similar fractions of conserved positions. DHSs, although also significantly enriched in conserved positions, are least conserved compared to all other functional elements. 5′ UTRs and CpG Islands display the highest level of conservation among putative regulatory regions, although generally sequence regions proximal to genes are more conserved.

### Fraction of Functional Positions across ENCODE Regions

What fraction of ENCODE regions are functional? If conservation is interpreted as a signal of selective constraint, SCONE may be employed to identify the number of functional positions in a genomic region (that is, a set of sites, not necessarily contiguous). The false discovery rate at a particular *p*-value threshold estimates the fraction of sites with *p*-values below that threshold that must be functional in order to explain the deviation from the expected neutral distribution. By relaxing the *p*-value threshold sufficiently, nearly all conserved functional sites may be included.

Based on the distribution of SCONE *p*-values and the false discovery rate, we estimate that between 5.5% and 11% of positions in ENCODE regions are conserved because of function. However, Figure 3 should not be taken as representative for the human genome as a whole, since ENCODE regions have a twice higher fraction of coding positions compared to the genome-wide average. In addition, stochastic variation in the mutation process and sampling variance inevitably introduce noise into the distribution, reducing the accuracy of the estimate.

### Clustering of Conserved Positions

A substantial fraction of conserved positions lies outside of MCS elements (65% at a SCONE *p*-value threshold for conservation <0.025, where the fraction of all ENCODE positions called conserved according to SCONE *p*-value or membership in MCS elements is approximately equal). Are these positions randomly distributed, or do they exhibit clustering, as we might expect if this observed sequence conservation is due to binding sites or other short functional elements? To evaluate the extent of clustering of conserved nucleotides, we identified all conserved islands (see Methods), using a *p*-value threshold of T = 0.05. These represent optimally bounded islands of conservation whose overall conservation cannot be increased by expanding them in either direction.

**Figure 4:**
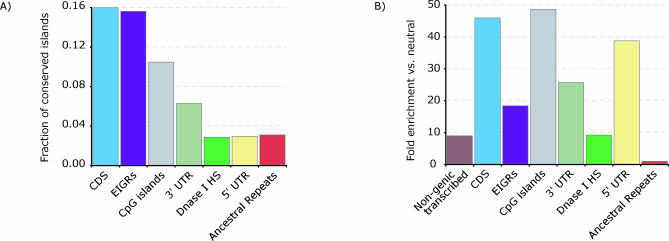
Islands of Conservation in Functionally Annotated Regions (A) Localization of short (5–12 bp) conserved islands in functionally annotated regions. Shown is the fraction of all islands that fall within a particular region. Nongenic transcribed regions were omitted to preserve scale, but contain 93% of short conserved islands. (B) Fold excess of short (5–12 bp) conserved islands in functionally annotated regions compared to ancestral repeat regions. Shown is the ratio of the density in each region (number of clusters divided by total number of positions in the region) to the density in ancestral repeat positions.

We restricted our analysis to short conserved islands, with lengths ranging from five to 12 bases. These islands are heavily overrepresented in functional sequences, with the majority found in and around coding sequences or promoter regions (Figure 4A).

We compared the density of short conserved islands (number of clusters/total number of positions) in functionally annotated regions to the density in putatively neutral ancestral repeat regions. As seen from Figure 4B, all functionally annotated regions show considerable over-representation of conserved islands with lengths ranging from five to 12 bases when compared to ancestral repeat regions. To verify that this is not simply due to a simple excess of conserved positions and is indeed due to significant clustering of conserved bases, we randomly shuffled SCONE *p*-value scores across all DHS sites and compared the proportion of short conserved islands predicted in these “shuffled” DHS positions; we find a 5-fold excess in density in unshuffled DHS sites, suggesting that the overrepresentation in functional regions is due to clustering. Stretches of two or three conserved bases do not show any over-representation in comparison to the shuffled sequence (unpublished data). In agreement with the results of the analysis of human genetic variation described above, over-representation of short stretches of conserved positions in functionally annotated regions suggests the functional importance of these short islands.

Can SCONE scores be used to identify isolated individual functional noncoding positions? In general, the rate of false discovery is too high across the study regions to allow ascertainment of such positions. However, in a subset of sites with either a higher expected mutation rate or greater sequence coverage, this may be possible. We chose a more stringent *p*-value threshold (*p* < 0.001); at this threshold, the computed false discovery rate in noncoding, non-MCS regions was 39%, meaning 61% of these positions are putatively functional. Based on the observation of enrichment of short conserved sequences, we looked for clusters of three non-MCS noncoding positions, each with a SCONE *p*-value < 0.001, that fell within a 10 bp window. We identified 5,562 such clusters in the study regions. A majority of them (80%) lie in CpG islands, as expected since conserved positions there will tend to have greater significance as determined by SCONE, due to their much higher mutation rate. These clusters are highly enriched amongst annotated regions such as DHS sites, showing a 520-fold increase in density compared to ancestral repeat regions and an 88-fold increase compared to regions lacking an annotation (that is, excluding exons, CpG islands, transcribed sequences, and DHS sites). Shuffling positions within DHS sites also greatly reduces enrichment (44 clusters identified within shuffled DHS positions compared to 1,079 within unshuffled positions). This enrichment is weaker, but still persists, even if we apply more stringent standards, restricting ourselves to positions with *p* < 0.001 that are at least 50 bp from the nearest MCS element or CpG island; clusters identified using these thresholds still show a 59-fold increase in density within DHS sites compared to AR regions, and a 10-fold increase compared to unannotated regions. Although further validation of these positions is difficult, the strong degree of enrichment in annotated regions suggests that these positions are highly likely to be conserved due to function.

## Discussion

Detailed knowledge of the structure of coding sequences makes them much more tractable to conservation analysis. The genetic code, by itself, imposes significant constraints on such sequences and provides us with a framework by which we may better understand them. A number of methods have been developed that exploit this knowledge to better predict functional and selective constraints on coding positions [5–7]. In coding regions, the functional significance of a given position is highly contingent upon the surrounding bases, since a protein, to some extent, behaves as a single coherent functional, and thus evolutionary, unit. The constraints imposed by this contingency means the influence of purifying selection on a site will be much easier to trace through its evolutionary history, since it is anchored by other sites that are similarly constrained. Finally, the existence of the genetic code dictates that the evolution of coding sequences is based almost wholly on their informational content.

In noncoding sequences, however, this situation does not persist. Few noncoding elements are as well-characterized in terms of structure and function as coding sequences are, but undoubtedly many elements will show markedly different patterns of evolution from what we have come to expect in coding sequences. These differences suggest that paradigmatic assumptions about conservation cannot be carried over from coding sequences, and conservation analysis in noncoding regions must proceed on fundamentally different grounds. In noncoding sequences, we might expect to find elements that, though highly functional, lack the same tight relationship between relative position and function. If, for example, the sequence composition bias of a region has a strong functional significance, the region might evolve to conserve the overall necessary property while showing very little constraint at individual sites. Structural properties of the sequence may show as much or more influence than informational content. Such properties may not depend on strict sequence conservation, and the evolutionary retention of such properties would therefore be invisible to traditional sequence conservation analysis.

Functional noncoding elements that show strong constraint might only be short, partially degenerate words only a handful of base pairs in length. Control of gene expression, for example, may depend on brief, influential segments (e.g., transcription factor binding sites) interspersed by relatively unimportant noise. Without the benefit of long contiguous stretches of functional positions to indicate important elements, identification of such words amidst the general rubble is considerably harder.

Our method attempts to take into account some of the above difficulties. SCONE is not based on any model of pattern of conservation along the sequence and is focused instead on individual nucleotide positions. Conservation of a specific nucleotide position does not directly depend on conservation of its neighbors. Along the same lines, when we score sequence regions, we use a purely additive scoring scheme. Additionally, SCONE optionally incorporates insertions and deletions. The importance of insertions and deletions for the analysis of conservation is probably greater in noncoding sequences than in genes, where most insertions and deletions will lead to frame-shifting mutations and thus are extremely rare.

Our analysis has revealed that many individually conserved nucleotides not embedded in conserved elements are of functional significance, as evident from analysis of the allele frequency distributions of human SNPs within these positions and analysis of individually conserved positions within functional features. These results suggest that future efforts at identifying functional positions in noncoding regions via analysis of conservation would benefit from methods that are hypothesis-free with respect to the distribution of constrained positions.

## Methods

For each nucleotide position, SCONE provides an estimate of the rate at which a site is evolving and the probability (*p*-value) that this position evolves neutrally given the rate estimate. We assess sequence conservation by implementing a model of sequence evolution. As is typical of most evolutionary models, the model implemented by SCONE treats nucleotide substitutions as a continuous-time Markov process. The SCONE model is more detailed, accounting for some of the influence of sequence context on nucleotide substitution rates, and optionally incorporates indel events. It requires as input a phylogenetic tree with known branch lengths (measured in substitutions per site rather than in years) and a multiple-sequence alignment of species within the given tree.

SCONE takes alignments as given, and does not attempt to make allowances for possible mistakes in the alignment process. Unaligned sequence is necessarily ignored, even though a subset of these positions might be conserved. Notably, misalignments may cause errors in reported positions, resulting in incorrect ascertainment of evolutionary rate. To minimize misalignments, we made use of alignments generated using TBA, a local (rather than a global) aligner, which emphasizes sensitivity rather than specificity.

We assumed that mutation processes are uniform across all species. This assumption breaks down once one descends below the mammalian family tree, because mutation patterns are believed to have changed at the time of mammalian radiation, especially in CpG dinucleotides [27], which affects both the mutation rate of those nucleotides and the frequency of CpGs in the genome. For this reason, we restrict all analysis performed with SCONE to mammals.

A second key assumption of our model is uniformity of mutation processes across the genome. Although there is a well-demonstrated heterogeneity in mutation rates even within chromosomes [28], the cause for this heterogeneity remains poorly understood and cannot be adequately modeled at this point. We instead assume a genome-wide average applies equally well in any context.

We consider only two kinds of mutations: substitutions and insertions/deletions (indels). The former are modeled as a continuous-time Markov process; the latter, comparatively rarer, are modeled as a linear process for convenience.

### Estimation of mutation rates.

A number of well-known parametric models for nucleotide substitutions exist [29]. Most of these do not consider the effect of sequence context on mutation rate, which can have profound effects on a site-specific measure of conservation. However, several recent studies derived context-dependent multiparametric models from deep mammalian phylogenies using diverse computational strategies [30–32].

An alternative strategy is to limit the analysis to very close genomes, where multiple substitutions per site are extremely rare and fully general mutation models can be derived using simple counting methods. Limiting the analysis to very close genomes avoids the complex problem of estimation of a very large number of parameters which arises in maximum likelihood or Bayesian methods which use deep phylogenies. We employed human–chimpanzee comparison to infer mutation rates. The substitution rate between human and chimpanzee is extremely low [33], so that the chance of observing double substitutions in the lineage at a particular site is negligible. At the same time, there is almost no incidence of shared polymorphism [33,34]. In the absence of multiple substitutions, transition matrix for nucleotide substitutions can be estimated by a simple counting approach. The ancestral state and the directionality of substitutions can be inferred by using baboon as an outgroup. As the divergence time between baboon and human/chimp is much greater, the probability of double substitutions is higher in this lineage. It is therefore necessary to correct for the occurrence of nonparsimonious situations.

To do so, we used a first-order model of dependence on neighboring positions in order to capture all context-dependent effects on mutation rate; a higher-order model would have been both unnecessary (unpublished data) and more computationally intensive. We made alignments of human, chimpanzee, and baboon sequence taken from ENCODE regions using the multiple sequence aligner TBA. The frequency of all trinucleotide triples within background positions in the aligned sequences was counted (ignoring any triples containing gaps). In order to correct for nonparsimonious situations, trinucleotide substitution rates in the human lineage, *p*(*A → B*), were computed in a manner similar to Jordan et al. [35].

This allows us to build a Markov transition rate matrix *Q*, where *Q*(*i,j*) = *p*(*i → j*), *i* ≠ *j*, and 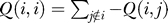 . If we operate under the assumption that the human lineage after divergence from chimp is short enough that multiple mutation events are unlikely at a single site, this transition rate matrix, if scaled to a unit time, will be identical to the instantaneous rate matrix for the human lineage. Under a standard continuous-time Markov model, we may compute a transition rate matrix *P* for an arbitrary time *t* according to the matrix exponential 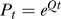 . We compute the matrix exponential using the Padé approximation [36].

Our estimate compares well against other previous estimates of context-dependent mutation rates [30–32]. Direct comparison shows our matrix is extremely similar (R^2^ = 0.96) to the matrix produced by Siepel and Haussler, while a matrix produced without context dependency, estimated via PAML [37] on the same data using the HKY85 model [38], fares much worse (R^2^ = 0.25).

### Gap model.

SCONE optionally allows inclusion of a model of insertion/deletion mutations in assessing conservation. Indel rates were estimated using a procedure similar to that for mutation rates. Four classes of insertions and deletions (of size 1, 2, 3, or greater than 3) were considered. Human/chimp/baboon alignments were used to infer the total number of insertion and deletion events of each class in the human lineage for the entire sample (with baboon as the outgroup to determine the ancestral state), as well as the number of ancestral bases, *N_a_*.

The frequency of an insertion of size *k* in a window of size *n* is ins(*k*)*n / *N_a_*, where ins(*k*) is the number of insertions of size *k* observed in the sample; the probability of seeing a deletion of size *k* at a single site is (*k + n −* 1)*del(*k*) / *N_a_*, where del(*k*) is the number of deletions of size *k* observed in the sample.

Indel events are comparatively rare, more than an order of magnitude less frequent than substitutions (Cooper et al., 2004). Thus, we assumed the absence of double-hit events on individual branches of the mammalian tree. The probability of an indel event therefore scales linearly according to time. Although this is not ideal, it avoids the considerable computational complications imposed by considering convolutions of indels.

### Computation of evolutionary rate.

SCONE requires as input a rooted phylogenetic tree for all species in an alignment intended to be scored, with branch lengths denoted in substitutions per site.

The algorithm proceeds in two phases: first, it computes an estimate of the rate of evolution of the site based on the observed alignment columns; then it computes a *p*-value for the rate score. The rate estimate is computed as follows.

Consider an alignment of *N* sequences and a column *i* of the alignment. Let *s*(*i,n*) be the “state” of the *n*-th sequence in the *i*-th column, where possible states are *S* = {A,T,G,C,1,2,3,4}, either nucleotide bases (A,T,G,C), or the size of an overlapping gap (1,2,3,4+). Then *c*(*i,n*) = (*s*(*i −* 1*,n*),s(*i,n*),*s*(*i* + 1,*n*)) describes the sequence context of the *i*-th column in the *n*-th sequence.

To compute the rate, we begin by labeling the leaves of a phylogenetic tree Ψ with the sequence state *c*(*i,n*) for all *n* ∈ *N* sequences (species) in the alignment.

We define the transition probability between two states *a* = (A*_i_*_−1_,A*_i_*,A*_i_*_+1_), *b* = (B*_i_*_−1_,B*_i_*,B*_i+_*_1_) as *p*(*a*,*b,t*) = subst(*a,b,t*)*indel(*a,b,t*), where subst(*a,b,t*) = *P_t_*(*a,b*) and indel(*a,b,t*) is the probability of the specific insertion or deletion event between *a* and *b* as derived from our gap model above (1 if we are ignoring gaps when computing rate) for time *t*.

We further define a conditional transition probability between two states *a, b* as:

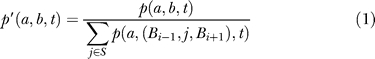
or, alternatively, if Ψ is a branch of length *t* in Ψ between nodes with states *a* and *b*, *p′*(Ψ) = *p′*(*a,b,t*).

Using this conditional probability, we compute the likelihood, recursively:

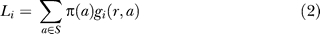
where π(*a*) is the probability of seeing state *a* and for a node *n* with children *n*_1_, *n*_2_:

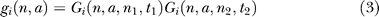


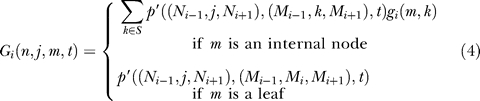



The state (*N*_i−1_ *,N,N_i+1_*) of node *n* is taken from the sequence, if *n* is a leaf; if *n* is an internal node, the values of *N_i_*_−*1*_ and *N_i+1_* are taken from the most parsimonious labeling of the tree at positions *i−*1 and *i+*1*.* Iteration thus proceeds over all possible states only for *N*, while *N_i_*_−*1*_ and *N_i+1_* are fixed.

*L* is the likelihood of the observed state under the assumption of neutrality. We introduce the factor *ω* to represent the rate of evolution of the site; this represents a scaling of the entire tree, not the individual branches of the tree. That is, in a site evolving at rate *ω*, all branch lengths in the given (neutral) tree are uniformly scaled by the factor *ω*. If *ω* < 1, the site is evolving slower than expected by neutrality, etc. The transition probability *p′* between states *a* and *b* for a site evolving at rate ω is *p′* (*a,b,ωt*), and similarly for *L*, e.g.,:

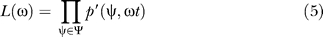



Note that *ω* is independent of the mutation rate at the site.

We may take two routes from this point. The simplest is to estimate *ω* by finding the value that maximizes *L* (by, for example, golden section search). The rate of evolution is the best possible discriminator we can use to distinguish between sites. However, our ability to accurately estimate *ω* depends on the mutation rate and depth of the tree at the site. For sites with very low mutation rate or very little sequence coverage, maximum-likelihood estimates of *ω* will be inaccurate due to sampling errors. A Bayesian estimate of *ω* may be more appropriate.

A number of Bayesian estimators and choice of prior distribution are available. SCONE allows the choice of multiple prior distributions, including a Gamma distribution and a log-normal distribution (or an arbitrary user-specified distribution). In the analysis presented here, rather than exploring these options exhaustively, we opted for simplicity and selected a relatively conservative estimator, *ω*, at the median value of the posterior probability distribution, and a simple prior, a uniform distribution [0,1].

SCONE allows either the Bayes estimate of *ω* or the maximum-likelihood estimate of *ω* to be reported. This rate estimate is called the “SCONE score” for the site.

### Computation of *p*-value.

Exact computation of a *p*-value for the above rate estimate is theoretically possible; however, enumerating across the space of all possible leaf states increases in computational cost exponentially as the number of species in the tree grows, and is usually prohibitively expensive. This computation is also apparently intractable to dynamic programming methods.

To avoid such costs, we approximate *p*-values via Monte Carlo simulation, which allows an arbitrary degree of precision. In practice, we usually perform 10^4^ iterations. For present purposes, this level of error is sufficient.

In each iteration, we perform a forward simulation of neutral evolution at a site. We first label the root of the phylogenetic tree with a random state *a* = (*i,j,k*), *i,j,k* ∈ {A,T,G,C}. Next, each daughter node *b* is labeled by random sampling according to the transition probability *p*(*a,b,t*), where *t* is the length of the intervening branch. This process is repeated until the entire tree is labeled. Special dispensations must be made for insertions, which propagate toward the root of the tree, symbolically. Therefore, insertion events are computed independently after the initial labeling, then propagated along the tree. Subsequently, the rate estimate is computed for the given labeling of the tree.

Sufficient iteration allows us to determine the approximate shape of the distribution of rates for the given phylogenetic tree. We may then approximate a *p*-value for a given rate estimate as the fraction of rates in the Monte Carlo sample that exceed the given rate. Iteration is performed once for each tree topology encountered—the resulting distribution is then memorized and used to compute *p*-values for subsequent positions with the same topology.

### False discovery rate.

We estimate the false discovery rate for a particular *p*-value threshold *p* as follows.

Under a simple model of evolution, we assume that some fraction *N* of all sites under consideration are evolving neutrally, while the remainder 1−*N* are functional. Furthermore, some fraction *C* of all sites will be scored by SCONE with a *p*-value below *p*. Because SCONE scores are uniformly distributed in neutral sites, the fraction of neutral positions with a *p*-value below *p* will be simply *p*. The fraction of functional positions with a *p*-value below *p* is the unknown true-positive rate *t*. Therefore *C* = *pN* + *t*(1−*N*). And *N* = (*C*−*t*)/(*p*−*t*).

We may observe some further constraints. If *C* < *p*, then *N* > 1, and so C ≥ *p*. Also, if *t* < *C*, then *N* < 0; therefore *t* ≥ *C*. Under these constraints, the false discovery rate *pN*/*C* has a maximum when *t* = 1. To estimate the minimal proportion of functional positions, we may conservatively assume that *t* = 1. The false discovery rate is thus conservatively given by:

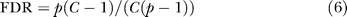
where *p* and *C* are known quantities. From this we may estimate both the false discovery rate and the proportion of functional sites in a genomic region.

### Missing information.

In any multiple sequence alignment of a given syntenic region, some species may show incomplete coverage, whether due to incomplete sequencing, alignment failure due to divergence, or differences in the evolutionary history of the site. We deal with missing information in several ways. If sequence information from certain species is missing from the alignment for whatever reason at a given site, then those species are pruned from the phylogenetic tree and SCONE scores are computed using the pruned tree for the site. If the tree has an empty root (that is, the site represents a recent insertion in the lineage of the reference sequence), scores are computed using the subset of the tree containing the insertion (as it makes little sense to track the evolution of a nonexistent site in the full tree).

### Mutation rate heterogeneity.

A major potential source of inaccuracies in the SCONE model is variation in mutation rate. Heterogeneity in mutation rate is well-documented [39] and is believed to vary at a scale somewhere between 1 Mb and 15 Mb [28]. As neutral divergence depends only on mutation rate [29], such heterogeneities are a potential source of model failure. Regions with lower mutation rate will tend to have an excess of apparently conserved positions, while regions with higher mutation rate will be depleted in conserved positions.

We quantified the deviation in SCONE *p*-value distributions for each sequence region as the mean *p*-value for that region (the expected mean for a [0,1] uniform distribution is exactly 0.5). We estimated mutation rate in non-CpG neutral positions for each region by counting the number of substitutions or indels per site between human and chimpanzee. According to these simple measures, deviation and mutation rate are significantly correlated (Figure S1C, R^2^ = 0.26). Some fraction of the deviation of SCONE *p*-value distributions from uniform expectation may thus be explained as the result of mutation rate variation between genomic regions. It is possible to partially correct for mutation rate effects simply by scaling the length of the phylogenetic tree according to local rate variation. Estimates of local mutation rates should be made using nucleotide divergence, which may lead to artificial correlations between mutation rate and sequence conservation, due to the influence of purifying selection on divergence rates. Though this correlation may be mitigated by making mutation rate estimates based on putatively neutrally evolving regions over a megabase scale, such estimates would require the input of external genomic annotations into SCONE. These corrections may be externally applied as seen fit; we have elected to avoid such heuristic corrections within our model. Future developments in the understanding of the causes of mutation rate heterogeneity will hopefully allow the construction of more precise mutation models.

### Comparison against existing scores.

A number of methods already exist that make use of multiple sequence alignments to score conservation, including several single-position scores [11,13,14]. SCONE is similar to previous single-position scores, in that it relies on a given phylogenetic tree and compares expected versus observed rates of evolution. Like GERP [11] and the parsimony-based *p*-value score available as part of the FootPrinter software package[13] (and unlike the phylogenetic shadowing method of Boffelli et al.), SCONE has no model for functional sequences, and instead is based on a null model of neutrality. Although SCONE may be run using the simple parsimonious substitution-counting method employed by Margulies et al., its intended mode relies on estimation of the rate of evolution of a site. SCONE differs from GERP and phylogenetic shadowing in that it computes a *p*-value for each position, in addition to making an estimate of the rate of evolution of the site. SCONE is the only method of scoring-sequence conservation that makes use of a context-dependent model of mutation.

We compared SCONE against several scores that were employed in generating the ENCODE MCS elements set (viz., phastCons [10], BinCons, and GERP). BinCons is a truly regional measure of sequence conservation, i.e., it scores conservation of sequence regions rather than individual nucleotides. PhastCons assigns scores in the form of Bayesian posterior probabilities to individual positions. However, these scores depend on the overall level of regional conservation. GERP scores conservation of individual nucleotide positions independently. We examined the utility of various conservation metrics in distinguishing between functional and neutral positions. We employed nondegenerate coding positions as the best available model of functional positions (although we are more broadly interested in applying SCONE to analysis of noncoding sites). Figure S2 represents ROC curves for all scores in terms of their ability to discriminate nondegenerate positions from ancestral repeat positions (Figure S2A) or 4-fold degenerate positions (Figure S2B).

From the ROC curves for each conservation score (Figure S2A), it is evident that regional measures of conservation, such as phastCons or BinCons, are able to return a more efficient partition of functional and nonfunctional sites than position-specific measures (GERP and SCONE), as regional scores rely on the interdependence of conservation amongst neighboring positions in functional elements. However, a similar comparison between positions showing heterogeneous conservation, using 4-fold degenerate coding positions as the “neutral” dataset (Figure S2B), paints a very different picture. Region-based conservation scores are less suited to distinguishing between contiguous conserved and nonconserved positions.

In comparing position-specific scores, SCONE is much more efficient at distinguishing nondegenerate coding and AR positions. This suggests that the context-dependent model employed by SCONE improves the identification of conserved positions. However, GERP more successfully discriminates nondegenerate coding positions from 4-fold degenerate coding positions. The difference between GERP and SCONE in this test is solely due to fully conserved positions and can be attributed to SCONE's context-dependent matrix; running SCONE with the Kimura two-parameter model recapitulates GERP's behavior. This discrepancy is unlikely to be explained by a difference in mutation rate patterns between synonymous and noncoding positions. Raw mutation rates and context-dependent effects (most notably CpG effect) are similar between coding and noncoding regions [40]. Thus, the most likely explanation is the well-documented effect of natural selection in favor of C and G in degenerate positions [41,42]. The top 1% of highly constrained sites according to SCONE are fully conserved positions within CpG dinucleotides, or positions within CpG di-nucleotides containing only a single substitution. The mutation rate for CpG dinucleotides is greatly elevated, and coding sequences are no exception. Such a high fraction of conserved CpG positions is extremely unexpected by chance and suggests they are maintained by selection. Another observation suggesting that conservation of these positions is due to selection is the clustering of these highly conserved 4-fold degenerate C and G nucleotides along the sequence. In 55% of cases, a 4-fold degenerate position neighboring a highly constrained 4-fold degenerate position is also conserved (SCONE *p*-value < 0.05).

### Scoring in ENCODE regions.

For the purposes of the analysis performed in this paper, SCONE scores were generated using Bayesian estimates of rate based on the most parsimonious labeling of the tree and including indels in the model. The phylogenetic tree was provided by the ENCODE MSA group. Human sequence was excluded from score generation; since SNP positions may appear in human consensus sequence and thus be counted as substitutions, significant artificial correlations between allele frequency and conservation may result from the inclusion of human sequence.

### Conserved elements.

Conserved elements are defined according to an additive score. For a given confidence threshold *T* and a series of bases numbered 1..N:

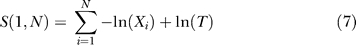



where *X_i_* is the SCONE score at position *i*. Here *T* represents the threshold of conservation for individual positions; relaxing this threshold results in larger contiguous elements being defined as conserved. A conserved element represents an optimally bounded region of sequence, for which the sum *S* cannot be increased by extension in either direction, which may be identified by an efficient linear search. We begin with starting position *j* = 1 and compute *S*(*j,j+k*) for *k = j*..*N*. Then *k_0_* is the first value of *k* > *j* where *S*(*j,j+k*) < 0, and 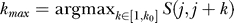 . For this interval, (*j*,*k_max_*) are the optimal bounds of a conserved element, with score *S*(*j*,*j*+*k_max_*). We define a new starting position *j* = *k_max_*+1 and continue iterating until we walk off the end of the sequence.

### Datasets.

Although there is currently no well-accepted annotation of strictly neutral regions in the human genome, and indeed each passing day seems to further whittle down those portions of the genome believed to be neutral, we made a best guess by excluding any features annotated as functional. A number of studies have employed mammalian ancestral repeats [13,18] as a neutral standard. These regions comprise repetitive elements that have been retained since the mammalian ancestor. We used these as our neutral standard and additionally excluded all sites falling within 50 bp of exon boundaries, regulatory regions identified by quantitative chromatin profiling [18], and CpG islands. This was the “neutral” set employed in all of our analysis.

We restricted ourselves to mammalian species in all our conservation analysis (excluding humans), which left 22 species in the ENCODE alignments, viz.: armadillo, baboon, chimp, colobus monkey, cow, dog, dusky titi, elephant, galago, hedgehog, human, macaque, marmoset, oppossum, mouse, mouse lemur, owl monkey, platypus, rabbit, rat, bat, shrew, and tenrec.

## Supporting Information

Figure S1 Properties of SCONE *p*-Values (505 KB PDF)

Figure S2 Comparison with Existing Methods (503 KB PDF)

## Abbreviations

DHS: DNase I hypersensitive sites
EIGR: experimentally identified genomic regions—regions with significantly increased histone modification
