# Supplementary material for: Analysis of Sequence Conservation at Nucleotide Resolution
Source: PLoS Comput Biol. 2007 Dec 28;3(12):e254. doi: 10.1371/journal.pcbi.0030254 (PMC2230682; doi:10.1371/journal.pcbi.0030254)
Supplement: Figure S1 — (505 KB PDF) [file pcbi.0030254.sg001.pdf]

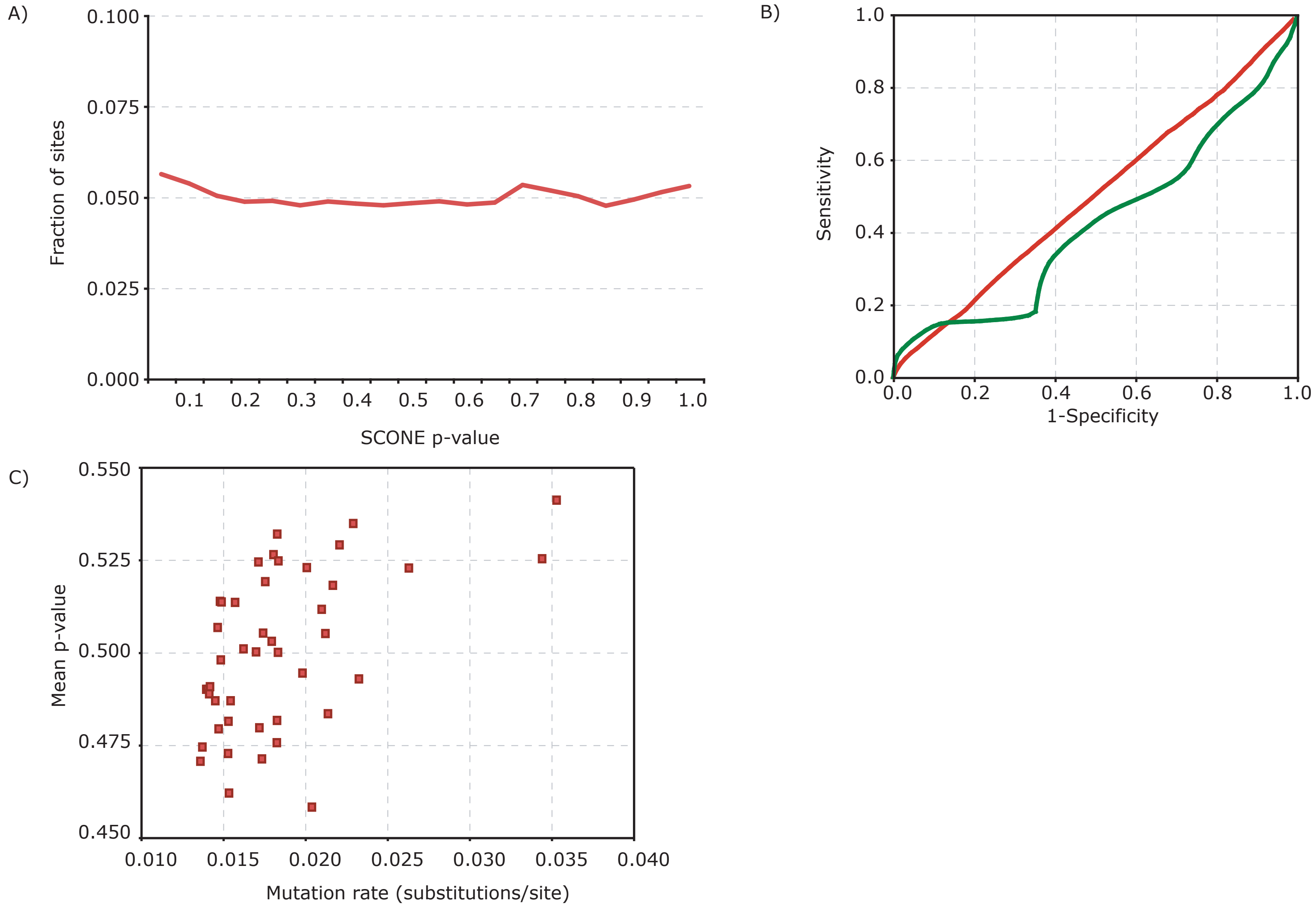

Figure S1. (a) Histogram of SCONE score p-values in neutral (ancestral repeat) positions. All histograms are represented as line plots for the sake of presentation. (b) ROC curve discriminating between high-coverage ancestral repeat regions and low-coverage ancestral repeat regions, using SCONE rate scores (green) and SCONE p-values (red), demonstrating that p-value scores are unbiased with respect to coverage; high- and low-coverage AR regions are considered comparably conserved by this measure. (c) SCONE score mean p-value vs. mutation rate (substitutions per site between human and chimp in ancestral repeats) for each ENCODE region. Correlation (R^2) between mean p-value and mutation rate is 0.26.
