# Supplementary material for: Analysis of Sequence Conservation at Nucleotide Resolution
Source: PLoS Comput Biol. 2007 Dec 28;3(12):e254. doi: 10.1371/journal.pcbi.0030254 (PMC2230682; doi:10.1371/journal.pcbi.0030254)
Supplement: Figure S2 — (503 KB PDF) [file pcbi.0030254.sg002.pdf]

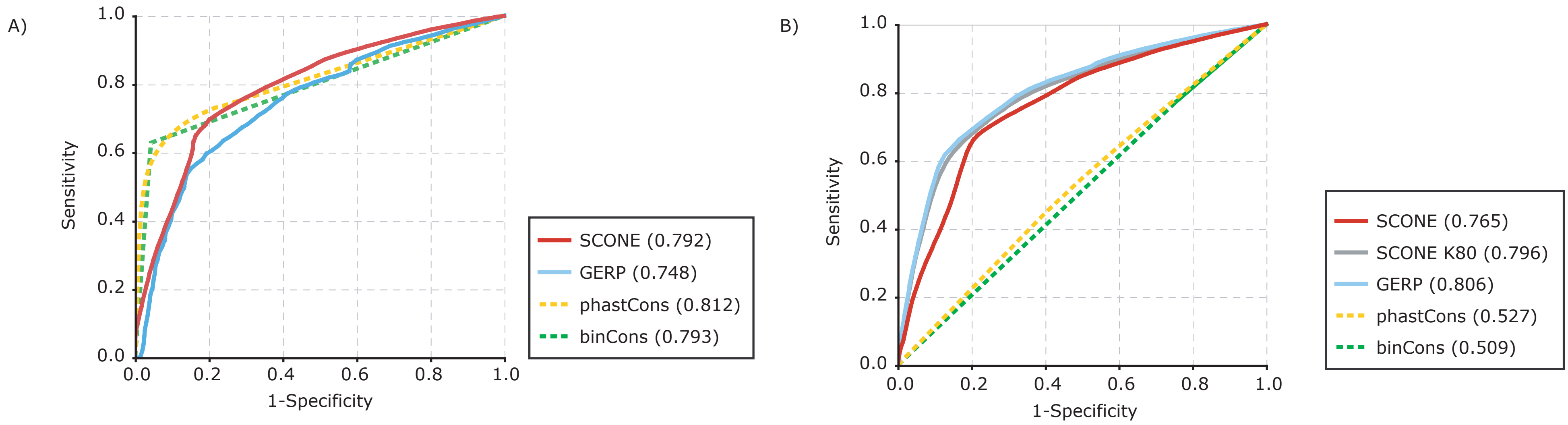

Figure S2. (a) ROC curves comparing partitioning of non-degenerate coding positions from ancestral repeat positions according to four different measures of conservation (phastCons, binCons, GERP, SCONE). AR regions and coding regions were selected for comparable levels of coverage to avoid biases due to lack of information. The area under the curve (AUC) is shown for each score in parentheses. (b) ROC curves comparing partitioning of non-degenerate coding positions from four-fold degenerate coding positions according to the above measures of conservation. Also included is an ROC curve for SCONE using a simple mutation (Kimura two-parameter model). AUC in parentheses.
